# Very delayed liver metastasis from small bowel gastrointestinal stromal tumor (32 years after resection of the small bowel GIST): Report of a case

**DOI:** 10.1016/j.ijscr.2020.09.155

**Published:** 2020-09-28

**Authors:** Masahiro Ishizaki, Futoshi Uno, Ryosuke Yoshida, Shunsaku Miyauchi, Osamu Honda

**Affiliations:** a Department of Surgery, Okayama Rosai Hospital, Okayama, Japan; b Department of Radiology, Okayama Rosai Hospital, Okayama, Japan

**Keywords:** GIST, Late liver metastasis, Follow up

## Abstract

**Introduction:**

Recurrent forms of gastrointestinal stromal tumor (GIST) include liver metastases and peritoneal dissemination. Recurrence often occurs within 2 years. We report a case of liver metastasis, which was detected 30 years after resection of a primary lesion in the small intestine and was resected 32 years later.

**Presentation of case:**

The patient was a 72-year-old woman and was being followed up for ureteral stones at the department of urology of our hospital. Computed tomography (CT) showed a small mass in segment 7 of the liver, 2 years ago. As the tumor gradually increased, a biopsy was performed, and a mesenchymal tumor was diagnosed. The tumor continued to increase in size and partial hepatectomy was performed. GIST was suspected from the sample extracted during hepatectomy. The patient had undergone a resection of the small intestine for a tumor 32 years ago. On tracing her medical records, it was confirmed that resection of the small intestine was performed for the diagnosis of leiomyosarcoma.

**Discussion:**

Based on the block specimen from 32 years ago, the tumor of the intestine was confirmed to be GIST, and the liver mass was finally diagnosed as liver metastasis of the GIST that had occurred 32 years ago.

**Conclusion:**

We experienced a case of liver metastasis 32 years after surgery for the first small intestinal GIST. To the best of our knowledge, this case had the longest disease-free interval before metastasis to the liver.

## Highlights

• This is a case with the longest disease free interval after GIST surgery before metastasis to the liver.
• Late liver metastasis can occur in the low risk group.
• Our case was in the low risk group as per the Modified-Fletcher classification.

## 1 Introduction

Gastrointestinal stromal tumors (GIST) are tumors derived from the interstitial cells of Cajal cells of the gastrointestinal tract [1], and were previously known as leiomyoma or leiomyosarcoma. Most postoperative recurrences occur within 2 years after surgery [2]. Metastatic disease is most commonly seen in the liver, at an average of 16–38 months after resection of the primary tumor [3,4].

We report a case of delayed liver metastasis of GIST, which was diagnosed as leiomyosarcoma of the small intestine 32 years ago. We also discuss other cases of delayed liver metastasis of GIST.

This paper has been reported in line with the SCARE criteria [5].

## 2 Presentation of case

**Fig. 1:**
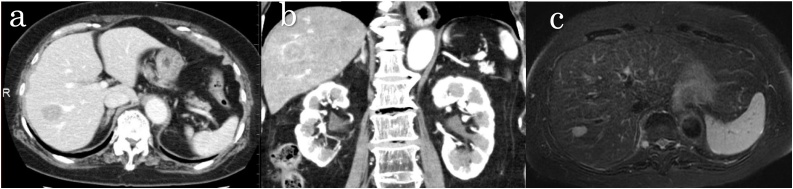
Axial (a) and coronal (b) contrast-enhanced CT images show a poorly enhancing mass in segment 7 of the liver. (c) Fat suppressed T2-weighted MR image shows slightly hyperintense mass in segment 7 of the liver.

**Fig. 2:**
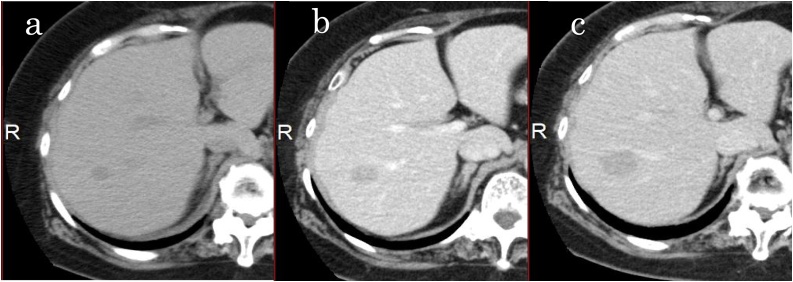
(a) The tumor in the S7 segment of the liver 2 years before resection (10 mm in diameter). (b) The tumor one year before liver resection (17 mm). (c) The tumor just before resection (24 mm).

A 72-year-old-woman with no symptoms was referred to our department for a tumor in the liver detected on CT-scan during the follow up for urinary stone. She had a history of resection of the small intestine 32 years ago for a tumor (Details of the disease were unknown). A slightly smaller lesion was seen in the previous CT scans taken at our hospital. Hemato-biochemical findings showed no abnormalities. There was no increase in the tumor markers for gastrointestinal cancer or hepatocellular carcinoma. Abdominal ultrasonography showed a tumor in segment 7 of the liver, which was hypoechoic but was difficult to diagnose on radiology. Abdominal contrast-enhanced CT showed a mass 2.4 cm in size with slight enhancement in segment 7 of the liver (Fig. 1). There were no findings suggestive of advanced cancer in the stomach or large intestine that could cause metastasis. Abdominal magnetic resonance imaging (MRI) showed hyperintense mass on diffusion-weighted imaging and a slightly hyperintense mass on fat-suppressed T2WI in segment 7 of the liver (Fig. 1). On re-evaluating the CT images taken at our institute in the past, we found that the lesions were not visible on the CT scan 4 years ago, but were slightly visible on the CT scans performed 2 years ago, and had gradually increased in size (Fig. 2).

Although HCC and metastatic liver cancer were suspected based on the above findings, there were no lesions suggestive of a primary lesion on gastroscopy and colonoscopy. Hence, a needle biopsy of the liver was performed, which revealed a mesenchymal lesion that is not usually seen in the liver.

**Fig. 3:**
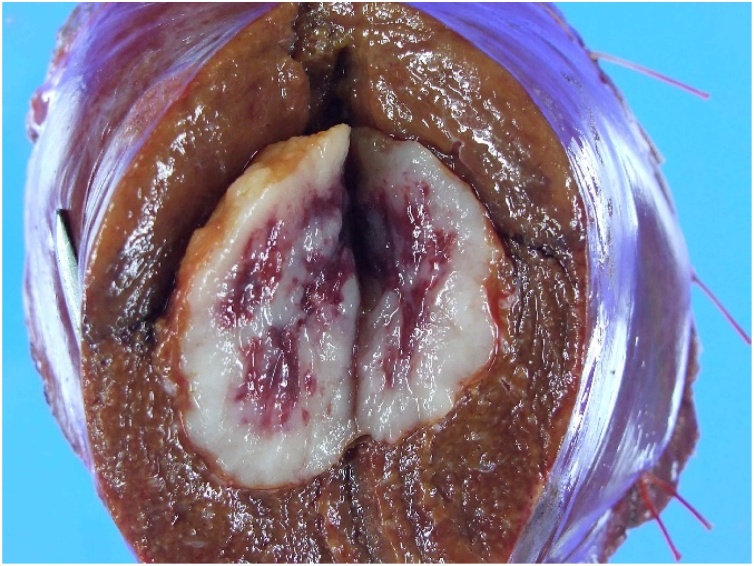
Macroscopic appearance of the resected specimen of the liver. The tumor is 25 × 25 × 20 mm in size.

**Fig. 4:**
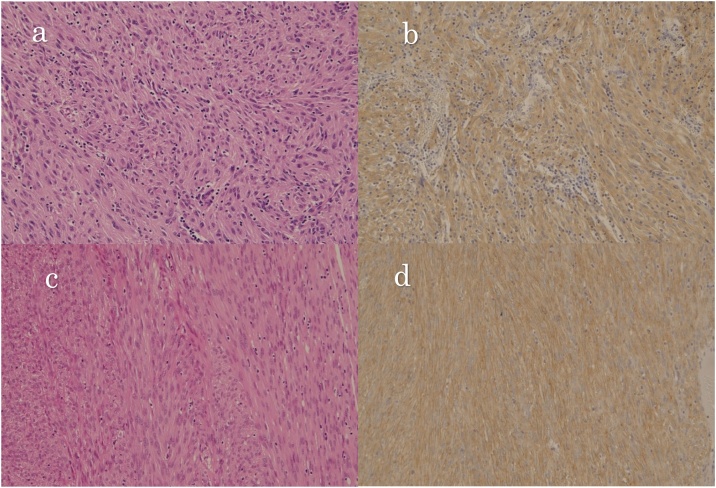
Microscopic findings. (a) HE stain (×200) of the tumor in the liver. (b) c-kit stain (×200). (c) HE stain (×200) of the tumor of small intestine 32 years ago. (d) c-kit stain (×200) of the tumor of the small intestine 32 years ago.

Therefore, a surgery was planned. The tumor was located in segment 7 of the liver, and partial resection of segment 7 of the liver was performed (Fig. 3). The post-operative course was uneventful. Pathological findings of the resected specimen showed a characteristic appearance of GIST with convoluted spindle-shaped tumor cells, and HCC and metastatic gastrointestinal cancer were ruled out. Immunohistochemical staining revealed CK7 (-), CK 20 (-), CAM 5.2 (-), Alpha-SMA (-), Vimentin (+), CD 34 (-), C-kit (+), Desmin (-) (Fig. 4).

Based on the above results, her past medical records were obtained from the institute where she was operated 32 years ago, and it was found that the small intestine was resected based on the diagnosis of a leiomyosarcoma of the small intestine, and the block specimen from that time was available. The results of the block specimen were compared with those of the resected lesion at our institute, and the results were almost identical. Since primary GIST in the liver is extremely rare and PET CT scan showed no other GIST lesion that could have caused liver metastasis, the lesion was finally diagnosed as liver metastasis of small intestinal GIST that occurred 32 years ago. Adjuvant therapy was also considered, but it was not administered at the patient's request, and the patient has been free from recurrence for 5 years.

## 3 Discussion

GISTs account for 0.2–0.5% of all tumors of the digestive tract and have the highest incidence (approximately 80%) among mesenchymal tumors [6,7]. These tumors are derived from the Cajal intercalated cells proposed by Rosai in 1996 [1], and have been attributed to function mutations in the c-kit and PDGFRA genes [7]. In addition to the stomach, small intestine, large intestine, and esophagus, non-gastrointestinal sites such as omentum and mesenteric retroperitoneum have been reported [8,9].

In this case, the possibility of GIST was suspected from the pathological analysis of the specimen obtained on biopsy of the liver lesion. However, primary GIST of liver has been rarely reported in the past [10,11]. An identical histopathological match with the specimen diagnosed as leiomyosarcoma 32 years ago, enabled us to diagnose liver metastasis of GIST. During the earlier times, there was no concept of GIST, and we were able to arrive at a diagnosis because the block of specimens from her previous surgery were available. The GIST had occurred 32 years ago, and the patient herself did not recognize the relationship with the tumor of small intestine.

Cases of liver metastases from gastrointestinal GIST more than 10 years later have sometimes been reported in the Japanese and other literature (Table 1) [[12], [13], [14], [15], [16], [17], [18], [19], [20], [21], [22], [23], [24], [25], [26]]. Although some cases of metastasis have been reported 20 years after the initial gastrointestinal surgery, our search revealed that this case had the longest disease free interval between resection of the primary tumor and resection of liver metastasis. The details of 16 cases of delayed metastasis were examined. The age at detection of the liver metastases was 58–84 years (median 66), 12 patients were males and 4 were females. The stomach was the most common site of the primary tumor, followed by the duodenum. The size of the primary lesion was more than 5 cm in 8 cases. Only 3 cases had a mitotic index of 10/50 or more, and 8 cases were classified as “High risk group” according to the Modified-Fletcher classification [1,27]. These cases indicate that late liver metastasis can occur in the low risk group. Our case was in the low risk group according to the Modified-Fletcher classification.

**Table 1:** Summary of Cases With Liver Metastasis Detected After a Lapse of More Than 10 Years From The Resection of Primary GIST.

author	year	age	sex	primary lesion	size of primary lesion (cm)	mitotic index	primary diagnosis	yeares to liver metastasis	risk of primary lesion (modified-Fletcher)	prognosis after hepatectomy (Y:year,M:month)
Balliani	1998	62	male	stomach	4	1/50	leiomyosarcoma	11	Low	–
Furukawa	2010	60	male	stomach	5	–	leiomyosarcoma	11	Intermediate	>1Y2M
Suito	2015	60	male	duodenum	10	3/50	psuedopapillary tumor	11	High	>3Y
Kishi	2019	77	male	duodenum	6.5	10/50	–	11	High	>2Y
Masuoka	2003	58	male	rectum	4	10/50	leiomyosarcoma	12	Intermediate	–
Yonezawa	2004	74	male	stomach	–	–	leiomyoma	12	–	>10M
Ueda	2012	80	male	stomach	–	–	unknown	12	–	>1Y
Kikuchi	2006	58	male	stomach	18	<1/50	leiomyosrcoma	13	High	>1Y
Miyamoto	2016	70	male	duodenum	5.5	3/50	unknown	13	High	>1Y4M
Tsuge	2008	56	female	stomach	5.8	–	leiomyosarcoma	15	Intermediate	–
Matsuoka	2007	55	female	retroperitoneum	14	–	leiomyosarcoma	17	High	>2Y6M
Uesaka	2017	84	female	small intestine	12	–	GIST	18	High	>7M
Omura	2017	72	male	stomach	3	–	leiomyoma	18	High	>1Y5M
Grossi	2017	79	male	stomach	–	10/50	benign leiomyoblastoma	23	High	>4Y
Ginori	2015	71	male	duodenum	2.5	1/50	shwannoma	29	Low	no hepatectomy
Our case	2020	72	female	small intestine	–	<1/50	leiomyosarcoma	32	Low	>5Y10M

Considering the long-interval before recurrence, the period for follow up of GIST after surgery needs to be determined. Japanese guideline [28] of GIST recommends the follow up within 10 years after resection of the primary tumor, even in the high risk group. However, considering the cases in Table 1, some cases of liver metastasis have been reported after ten years; patients should be informed about the possibility of late or very delayed recurrence even after ten years.

In this case, adjuvant chemotherapy after resection of the hepatic metastasis was not performed because of the patient's reluctance. Nunobe et al. [29] reported that the prognosis of patients with liver metastasis when the interval before recurrence was more than 5 years was better than that of patients in whom recurrence occurred in less than 5 years. The Japanese guidelines do not specifically recommend postoperative adjuvant therapy in such cases; hence, adjuvant chemotherapy was not administered. In fact, five patients in Table 1 were alive without recurrence for more than 2 years, and at least early hepatic recurrence within 6 months was not described in any of these articles. Thus the prognosis appears to be relatively good after resection of liver recurrence after more than 10 years. Our patient has been well without liver recurrence for more than five years.

## 4 Conclusion

We experienced a case of liver metastasis 32 years after surgery for the first small intestinal GIST. Recurrence of late liver metastasis can occur sometimes, and it is necessary for the attending physician to be aware of it and to educate the patient accordingly.

## Declaration of Competing Interest

The authors report no declarations of interest.

## Funding

No source of funding.

## Ethical approval

We have reported a single case, not a clinical study, with no requirement for ethical approval.

## Consent

Written informed consent was obtained from the patient for publication of this case report and accompanying images. A copy of the written consent is available for review by the Editor-in-Chief of this journal on request.

## Author contribution

Dr Masahiro Ishizaki: Investigation, Writing-original draft, Writing-Review and Editing, Visualization.

Dr Futoshi Uno: Review and Editing.

Dr Ryosuke Yoshida: Review and Editing.

Dr Shunsaku Miyauchi: Collecting datas, Review and Editing.

Dr Osamu Honda: Review and Editing about radiological materials.

## Registration of research studies

Not applicable.

## Guarantor

Dr Masahiro Ishizaki.

## Provenance and peer review

Not commissioned, externally peer-reviewed.
